# Neutralizing Anti-Hemagglutinin Monoclonal Antibodies Induced by Gene-Based Transfer Have Prophylactic and Therapeutic Effects on Influenza Virus Infection

**DOI:** 10.3390/vaccines6030035

**Published:** 2018-06-26

**Authors:** Tatsuya Yamazaki, Joe Chiba, Sachiko Akashi-Takamura

**Affiliations:** 1 Department of Microbiology and Immunology, Aichi Medical University, School of Medicine, 1-1 Yazakokarimata, Nagakute, Aichi, Tokyo 480-1195, Japan; yamazaki13@aichi-med-u.ac.jp; 2 Department of Biological Science and Technology, Tokyo University of Science, Niijuku 6-3-1, Katsushika-ku, Tokyo 125-8585, Japan; chibaj@rs.noda.tus.ac.jp

**Keywords:** hemagglutinin, antibody drug, passive immune-prophylaxis, passive immune-therapy, antibody-gene, gene therapy, plasmid vector, adeno-associated virus (AAV) vector, electro-transfer, hydrodynamic injection

## Abstract

Hemagglutinin (HA) of influenza virus is a major target for vaccines. HA initiates the internalization of the virus into the host cell by binding to host sialic acid receptors; therefore, inhibition of HA can significantly prevent influenza virus infection. However, the high diversity of HA permits the influenza virus to escape from host immunity. Moreover, the vaccine efficacy is poor in some high-risk populations (e.g., elderly or immunocompromised patients). Passive immunization with anti-HA monoclonal antibodies (mAbs) is an attractive therapy; however, this method has high production costs and requires repeated inoculations. To address these issues, several methods for long-term expression of mAb against influenza virus have been developed. Here, we provide an overview of methods using plasmid and viral adeno-associated virus (AAV) vectors that have been modified for higher expression of neutralizing antibodies in the host. We also examine two methods of injection, electro-transfer and hydrodynamic injection. Our results show that antibody gene transfer is effective against influenza virus infection even in immunocompromised mice, and antibody expression was detected in the serum and upper respiratory tract. We also demonstrate this method to be effective following influenza virus infection. Finally, we discuss the perspective of passive immunization with antibody gene transfer for future clinical trials.

## 1. Introduction

The influenza virus continues to be a major public health concern, causing annual epidemics and occasional pandemics. For prophylaxis, the first candidate is vaccination, which resulted in the eradication of smallpox and the drastic reduction of poliomyelitis [1]. The main target for influenza vaccine is hemagglutinin (HA), a membrane protein of the influenza virus. HA binds to the cell-surface sialic acid of host cells and the virus invades the host cell by endocytosis triggered by this binding. Antibodies against HA are effective against the virus because they prevent its binding to the host cells. However, the conventional influenza vaccine has had limited success due to antigenic drift and shift of viral proteins (e.g., HA) [2,3] and is dependent on the vaccinated individual [4] to induce potent long-term neutralizing antibodies. Moreover, it has been reported that the antibody response against the influenza virus in the elderly is considerably lower than in younger adults [5]. Because induction of protective immune responses by influenza vaccination requires 1–2 weeks, the patient will continue to be susceptible to viral infection after receiving the vaccine. The vaccine also has limited efficacy for other high-risk patients, including immunocompromised patients, organ transplant recipients, and patients suffering from autoimmune diseases [6,7,8,9,10,11]. 

Passive immuno-prophylaxis and immunotherapy may provide possible solutions to these problems. Inoculation with neutralizing monoclonal antibodies (mAbs) is expected to induce a rapid and potent protective effect independent of the individual’s immunocompetency [10]. This approach plays a key role in the protection against pathogenic infections, with a long history dating back to the production of anti-tetanus and anti-diphtheria serum in the late 19th century by Kitasato and Behring [12]. This research proved that neutralizing antibody inoculation has the potent ability to neutralize the pathogen. 

Now, it is known that some influenza virus antigens are conserved among strains [13]. Therefore, neutralizing mAbs that bind such antigens have been cloned and their characteristics have been reported by many researchers [14]. These mAbs have been shown to broadly protect against influenza virus infection; however, production costs are high and repetitive injections are required for this method to be effective. Because the half-life of human IgG is approximately 20 days [15], and that of mouse is normally 7 days [16], weekly or biweekly infusions of mAbs are required to prevent infection for prolonged period of time [17]. Because of this, the development of an “antibody drug” for prevention of infectious disease has been extremely challenging. 

In vivo antibody gene transfer using plasmids or viral vectors (e.g., adeno-associated virus (AAV)) is one approach that has been developed to address this problem. In a recent review, Hollevoet et al. reported that the antibody gene transfer has been used for more than 20 years [17,18]. In 1995, the first peer-reviewed pre-clinical study was reported regarding an adenoviral vector coding antibody gene against HER2 (erbB2), which is a known oncoprotein and a target for breast cancer [17,18]. Through this approach, long-term neutralizing antibodies are produced by the host cells with only a single administration [17,19]. Gene-based mAbs are also cost-effective in terms of production, purification, and administration [17,19]. We have previously generated a plasmid encoding neutralizing mAbs against the HA of A/Puerto Rico/8/34 (A/PR8; H1N1) influenza virus [20]. We were able to induce potent and long-term expression of the mAbs in mouse serum [20,21]. All mice that received the plasmid encoding the neutralizing mAbs showed long-term prophylaxis and even therapeutic treatment of influenza [20,21]. Here, we will review a novel passive immunization method using antibody-gene transfer to protect against influenza virus infection and discuss perspectives for clinical applications. 

## 2. Passive Immunization for Influenza by the Antibody Gene Transfer Method

### 2.1. HA Is the Main Target to Protect Against Influenza Virus Infection 

HA is a membrane protein of the influenza virus. HA primarily binds the host cell-surface receptor, 5-*N*-acetylneuraminic acid (sialic acid), which is attached to membrane glycoproteins, glycophospholipids, and proteoglycans [22,23]. It is the main target for vaccines and conventional passive immunization [24,25,26], because antibody blocking prevents virus-to-cell binding. However, the influenza virus continues to escape from host immunity (e.g., neutralizing antibodies), because the antigenicity of HA is different among virus strains, which is referred to as antigenic drift or shift [27,28]. HA consists of two domains. One is the head domain (HA1), which contains a highly variable amino acid sequence that allows the virus to evade the host’s immune system. The second domain is a stalk domain containing a transmembrane domain (HA2) which is highly conserved and therefore, a target for neutralization of the virus [13,26,29]. Recently, however, a conserved region in head domain called the “lateral patch” was identified and is being considered as a vaccine target [30]. Broadly reactive mAbs that bind the conserved region of HA have been developed as the result of multiple studies [14]. 

### 2.2. Long and Potent Expression of Neutralizing Antibodies by the Antibody Gene Transfer Method in Serum

**Figure 1:**
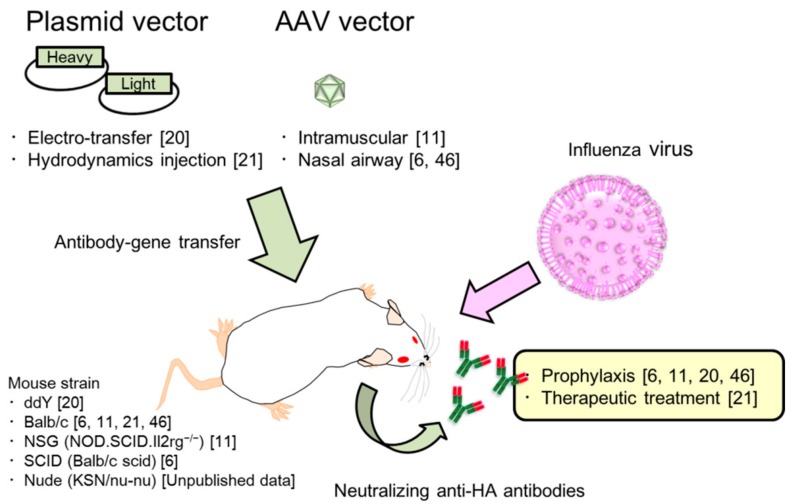
Strategy for the protection against influenza by passive-immunotherapy with antibody gene transfer.

The therapeutic antibody drug market has greatly increased since 1983, when the first clinical trial was initiated with mouse mAbs targeting the CD3 receptor [17,31]. Many approved antibody drugs are currently used to treat cancer, autoimmunity, and inflammation; however very few have been effective against infectious disease. However, the high costs that are associated with the production, purification, and quality control prevent the development of clinical mAbs against influenza. Moreover, long-term protection is difficult with a single inoculation because of the short half-life of the mAb. To resolve the problems associated with therapeutic antibody drugs, we firstly demonstrated passive immune-prophylaxis using the antibody gene transfer method against influenza virus infection (Figure 1). We performed electro-transfer of the plasmid encoding for neutralizing anti-HA mAb in mouse muscles which were pre-treated with hyaluronidase to enhance gene expression [20,32]. The antibody levels reached over 10 µg/mL in the serum and were stably maintained for at least 70 days after inoculation [20]. Moreover, the protective effect was retained for at least 130 days after the gene transfer. 

Using an AAV-based vector, Balazs et al. demonstrated the potent and stable expression of human anti-HA mAbs, which could broadly neutralize against multiple influenza virus strains [11]. Surprisingly, only a single inoculation induced around 50–200 µg/mL of mAb and this level was maintained for 448 days after the gene transfer, even in immunocompetent Balb/c mice [11]. These results suggest that the antibody gene was stably maintained in the host tissue and resulted in the potent and long-term expression of mAbs in the body. Additionally, this method can induce long-term prophylaxis against influenza virus infection by only a single inoculation. 

### 2.3. Two Kinds of Injection Methods, Electro-Transfer and Hydrodynamics Injection

**Figure 2:**
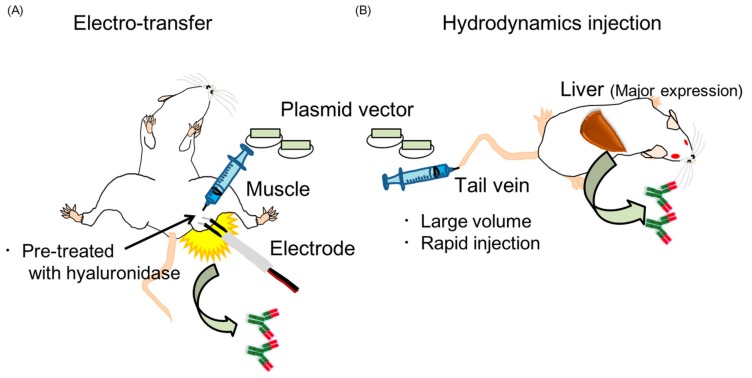
Scheme of electro-transfer (**A**) and hydrodynamics injection (**B**).

Here, we will describe the advantages of plasmid vectors and the mechanism of the long-expression by antibody gene transfer. We generated a plasmid encoding the genes for the heavy chain (IgG) or the light chain (kappa) of anti-HA antibody. Plasmid vectors are considered to be safer because they have been used in several clinical studies [33,34] and it has been demonstrated that the potential for integration into the host genome was negligible and the frequency was below the spontaneous mutation rate that occurs naturally in mammalian genomes [35,36]. Moreover, plasmid DNA does not induce an immune response against itself [35]. Plasmid vectors are also easy to prepare, stable during storage [37], and relatively inexpensive to produce. However, gene transfer with only needle-injection of the plasmid does not induce significant expression of the exogenous gene. The electro-transfer method (Figure 2A) is superior to injection only and is used for clinical trials [33,34,38,39,40]. As described above, we conducted electro-transfer in mice (Figure 2A) and successfully induced over 10 µg/mL of the antibodies in serum for at least 70 days following antibody gene transfer [20]. The expression levels significantly higher than the level of HA-specific IgG antibody induced from vaccination (1–3 µg/mL) in a previous report [41]. One of the reasons for the long-term expression is that plasmid DNA is taken up by skeletal muscle cells and retained long-term in an extrachromosomal, non-replicative circular form [42]. Danko et al. demonstrated the presence of the plasmid as foreign DNA in muscle for at least 30 days by Southern blotting [42]. They also confirmed the long-term retention of foreign DNA in muscle using a luciferase expression vector. The luciferase vector was injected into the muscle and luciferase activity was periodically measured. Luciferase expression could be detected for at least 60 days. From this result, they concluded that the foreign DNA was retained in the muscle long-term, because the half-life of luciferase protein and the in vitro RNA transcript appears to be less than 24 h. 

Here, we will also review another method of gene transfer, i.e., hydrodynamics injection (Figure 2B). In 1999, Liu and colleague demonstrated an innovative gene transfer method by hydrodynamics injection involving the rapid injection of a large volume of plasmid-DNA solution into mice through the tail vein [43]. This methods targets the liver and the peak expression was reached within 8 h after the gene transfer [43]. A remarkable study by Kitaguchi et al. indicated that the hydrodynamic injection of a plasmid encoding antibody genes could induce higher expression levels of the antibody than the electro-transfer method [44]. We confirmed this result within 5 days following the antibody gene transfer. We detected expression of the neutralizing antibody in the serum only 4 h after hydrodynamic injection, followed by peak expression of over 10 µg/mL [21]. However, Kitaguchi et al. also demonstrated that the expression level by hydrodynamic injection rapidly decreased after 40 days following gene transfer, while gene expression by electro-transfer was stable for at least 100 days. Therefore, hydrodynamics and electro-transfer are considered the best methods for short-term therapeutic treatment and long-term prophylaxis, respectively, against influenza virus infection.

### 2.4. Characterization of Antibody Gene Transfer with AAV Vector 

Viral vectors, such as adenoviral vector or AAV induce specific immune responses to itself, thereby limiting their efficacy. The human population has been naturally exposed to AAVs, resulting in the induction of specific neutralizing antibodies [36]. The immune response markedly limits the gene delivery by the AAV vector. Using a total of 226 donors between the ages of 25 and 64 years, Boutin et al. determined the ratio of prevalence of AAV by measuring the specific total IgG in healthy volunteers [45]. The results indicated that AAV1 and AAV2 were higher (67% and 72%, respectively) than AAV5 (40%), AAV6 (46%), AAV8 (38%), AAV9 (47%), and the two serotypes AAV2 (59%) and AAV1 (50.5%) had the highest neutralizing abilities and AAV8 (19%) and AAV5 (3.2%) had the lowest [45]. From these results, they recommend AAV5, AAV8, and AAV9 for gene therapy in the human population [45]. Studies using an AAV8 or AAV9-based vector encoding the antibody gene have shown long-term protective efficacy for influenza [6,11,46]. AAV is a nonpathogenic virus belonging to the parvovirus family and has a single-stranded DNA (ssDNA) genome [47,48]. Because AAV generally requires the helper virus, adenovirus, for a productive infection [48], recombinant AAV (rAAV) is prepared from packaging cells co-transfected with inverted terminal repeats (ITRs) flanking the transgene and the plasmid coding rep/cap gene of AAV, and the necessary adenovirus helper genes [49,50]. Moreover, self-complementary AAV (scAAV) was modified from rAAV and has the ability to re-fold into double stranded DNA templates for expression [48]. scAAV has been shown to induce more rapid and potent expression of the transgene [49]. It is suggested that scAAV can transduce muscle cells at least 10- to 15-fold more efficiently [49]. scAAV based vectors encoding antibody genes can stably induce potent levels of neutralizing antibodies (10–100 µg/mL) for over one year [11,51]. Therefore, the AAV vector is considered a powerful method of passive immunization with antibody genes. 

### 2.5. Modification of the Expression Vector for the Increased Expression of Neutralizing Antibodies 

**Figure 3:**
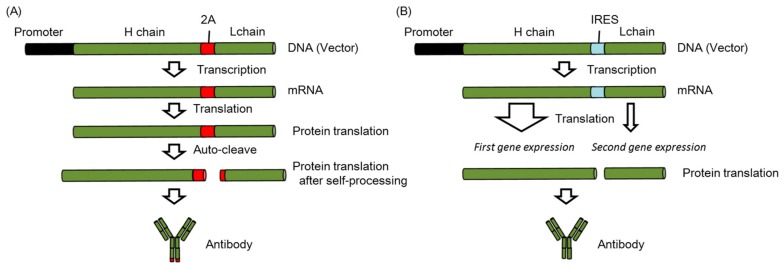
Antibody expression cassettes (**A**) The 2A peptide links the heavy (H) chain and light (L); (**B**) IRES links H chain and L chain.

To obtain higher expression of the mAb, Kitaguchi et al. previously modified the antibody expression cassettes [44]. They showed that the highest expression was from a plasmid encoding genes for both the light and heavy chains, followed by two separate plasmids encoding either the light or heavy chain gene. They also demonstrated that expression using a single promoter (the CMV) was 5-fold higher than with different promoters (the CMV and EF-1) [44]. Moreover, Fang et al. indicated that stable antibody expression at therapeutic levels could be maintained using the 2A peptide sequence located between heavy chain gene and light chain [50] (Figure 3A). The 2A peptide is derived from the foot-and-mouth-disease virus and can undergo self-cleavage to generate two proteins, which are full-length antibodies from a single open reading frame [50]. They indicated that the AAV vector encoding the antibody gene that included the 2A peptide could induce 16-fold higher mAb expression than the vector that included internal ribosomal entry sites (IRES) [50], in which the second gene expression was significantly lower than the first gene expression [52] (Figure 3B). 

### 2.6. Induction of Neutralizing Anti-HA Antibodies in the Upper Respiratory Tract by Antibody Gene Transfer 

Influenza virus initially infects and replicates in the epithelial cells on the upper respiratory tract, which is also the site of the first defense in influenza virus infection [53]. Therefore, we evaluated the reduction of virus titer in upper respiratory tract with neutralizing anti-HA IgG antibodies by electro-transfer and hydrodynamic injection of the antibody gene [20,21]. Antibodies were detected in the nasal wash obtained from the mice that were transferred with antibody gene [20,21]. It has been suggested that anti-HA IgG can also contribute to the prevention of infection in the upper respiratory tracts. Tamura et al. have also reported that IgG diffuses from the serum to the mucosal surface of the respiratory tract, which contributes to prevent influenza virus infection on the alveolar epithelia [54]. FcRn also efficiently contributes mucosal vaccination by transporting IgG over the mucosal epithelium and into the lumen of the lung, intestine, or vagina [16]. Therefore, it has possible that FcRn also plays an important role in passive immunization for influenza. 

On the other hand, it is well known from vaccine studies that the secretory IgA against influenza virus mainly protects the mucosal sites in the upper respiratory tract [4,53,54]. We were also able to induce neutralizing anti-HA IgA in the nasal mucosa with the antibody gene transfer by hydrodynamic injection [21]. Because the anti-HA IgA antibodies in the nasal wash could be bound to secretary components, they were likely transferred from the basolateral side to the apical surface by transcytosis via the polymeric Ig receptor (pIgR) [21,55]. Secretory IgA antibodies mainly contribute to humoral mucosal immunity to influenza virus infection [4,54,56]. Therefore, it is expected that the expression of secretary anti-HA IgA the upper respiratory tract plays a significant role in the prevention of influenza virus infection.

Notable research has indicated that the exogenous protein is directly induced in upper respiratory tract using the AAV9-based vector [6,46]. One study indicated that the expression level increased until 5 days after the gene transfer, followed by stable expression for at least 14 days [46]. Therefore, the AAV vector may be useful to directly induce neutralizing antibodies into mucosal membrane. 

### 2.7. Passive Prophylaxis for Immunocompetent and Immunocompromised Mice Using Antibody Gene Transfer

Next, we evaluated the potent prophylactic efficacy of the electro-transfer of the plasmid encoding neutralizing anti-HA mAbs against IAV infection [20]. By a single inoculation of 30 µg of plasmid in three muscles of immunocompetent mice (ddY mice), the expression levels reached a peak of over 10 µg/mL 20 days after the antibody gene transfer. The mice were then challenged with a lethal dose of influenza virus in the lower respiratory tract, with the lung as the main target. The viral titer in bronchoalveolar lavage was at undetectable levels for almost all challenged mice. Moreover, all the mice that received the antibody gene transfer survived for more than 14 days with almost no loss in body weight. We confirmed this protective effect for at least 130 days after the transfer. 

**Figure 4:**
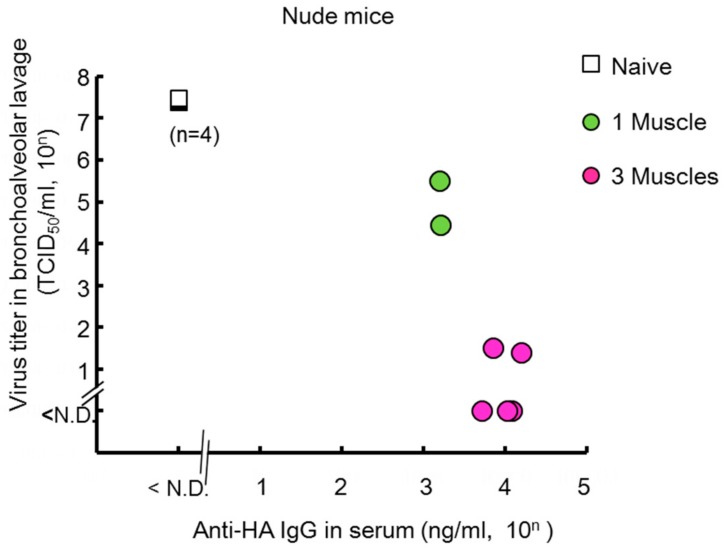
Long-prophylaxis against influenza virus infection in KSN/nu-nu nude mice that received electro-transfer of the plasmid encoding the neutralizing anti-HA mAb. The mice were challenged with 1000 TCID_50_/20 µL of influenza virus (A/PR8) 130 days after the gene transfer as indicated. At 3 days post-infection, the serum and bronchoalveolar lavage specimens were obtained. The expression level of the anti-HA antibodies in serum and the viral titer were measured. N.D., not detected. Data were analyzed using a non-parametric Kruskal–Wallis test (Virus titer: *p* = 0.0013, Anti-HA IgG: *p* = 0.0013).

As shown in previous paragraph, electro-transfer is effective in normal mice. A pre-clinical study has evaluated the efficacy of anti-influenza enriched hyperimmune intravenous immunoglobulin (IVIG) using SCID mice, which have severe immunodeficiency due to lack of functional B and T lymphocytes [57]. This research suggests that passive immunization is an effective strategy for use in immunocompromised patients. Balazs et al. demonstrated that intramuscular inoculation of AAV9 vector encoding the broadly neutralizing influenza antibody induced a prophylactic effect against a lethal dose of influenza virus in young mice (between 14 and 19 weeks of age), old mice (between 46 and 55 weeks of age), and NOD.SCID.Il2rg^−/−^ (NSG) mice that lack T cells, B cells, and functional NK cells [11,58]. Adams et al. also showed that airway inoculation with the AAV9 vector protected young, old, and immunocompromised (SCID) mice [6]. Using electro-transfer with our non-viral plasmid vector, we also determined that long-term protection was induced in nude (nu/nu) mice (unpublished data), which have a lower immune response against the influenza virus than normal mice [59,60]. The concentration of neutralizing antibodies in the serum reached approximately 10^3^ or 10^4^ ng/mL 130 days after the electro-transfer of the antibody gene into one muscle or three muscles (Figure 4). The viral titer in in bronchoalveolar lavage was reduced approximately 10^2^- or 10^7^-fold compared to naive mice (Figure 4). This research suggests that prophylactic approaches using antibody gene transfer may be effective in the protection of immunocompromised or elderly patient populations who currently receive limited protection from existing vaccines. 

### 2.8. Passive Therapeutic Treatment Against Influenza Virus Infection by Antibody Gene Transfer

**Figure 5:**
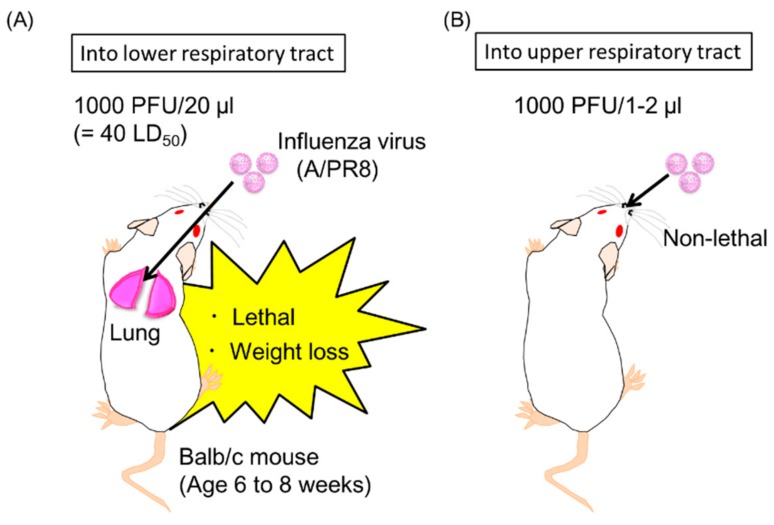
Mouse models of the influenza virus (A/PR8) infection [62]. (**A**) Into lower respiratory tract; (**B**) Into upper respiratory tract.

In addition to prophylaxis, the therapeutic treatment of influenza is also important for the control of the infection. The development of antiviral drugs, such as oseltamivir (Tamiflu), is essential for the therapeutic treatment of influenza; however, antigenic drift and shift often limit the efficacy as the viruses acquire resistance [61]. Therefore, we evaluated whether the antibody gene transfer could provide therapeutic treatment against established influenza virus infection. According to a modification of a procedure by Yetter et al., there are two mouse models of the influenza virus (A/PR8) infection (Figure 5) [62]. One is a lethal infection of the lower respiratory tract (Figure 5A), and the other is a non-lethal infection into the upper respiratory tract (Figure 5B). We demonstrated that hydrodynamic injection with the plasmid encoding the neutralizing anti-HA IgG antibody could induce protective effects as late as 2 days after lethal dose of influenza virus infection in lower respiratory tract (Figure 5A) [21]. The viral titer in the bronchoalveolar lavage was reduced to approximately 1/400 compared to the control group [21]. The hydrodynamic injection could also induce rapid and potent levels of the neutralizing antibodies in the serum. On the other hand, electro-transfer with the antibody gene failed to induce protective effects (unpublished data). To our knowledge, this is the first successful therapeutic treatment of influenza using antibody gene by hydrodynamic injection.

To treat influenza virus infection in the upper respiratory tract, we also generated anti-HA IgA, IgM, IgE, and IgD antibodies from the original anti-HA IgG antibody gene because there are great differences in the immunological functions of each antibody isotype [21]. We first generated the antibody gene expressing the anti-HA IgA, because several studies have demonstrated the prevention of influenza virus infection in the upper respiratory tract by passive intravenous injection of secretory IgA, but not IgG [63,64,65]. Secondly, we expected that anti-HA IgM had a possible role in the therapeutic treatment of influenza virus infection in the upper respiratory tract because joining chain positive IgM can be transported to the apical side of mucosal cells via a pIgR [66]. Finally, we generated the antibody gene of anti-HA IgE and IgD. IgE binds with the high-affinity IgE receptor (FcεRI) of mast cells and basophils and shows protective functions against parasites [67]. Recent research has demonstrated a new passive immunization method with anti-tumor IgE [68]. This study indicated that cross-presentation conducted by dendritic cells bound with anti-tumor IgE via high-affinity FcεRI mediated a cytotoxic T cell response followed by an anti-tumor immune response [68]. Therefore, it would be expected that anti-HA IgE could induce a potent immune response and be a potential therapeutic treatment against influenza virus infection. On the other hand, a previous report indicated that IgD production occurs in the upper respiratory mucosa, in which IgD recognizes respiratory bacteria [69]. Although the function of IgD in this context remains to be elucidated, IgD-stimulated basophils have been shown to produce antimicrobial factors that reduce bacterial growth [69]. Therefore, anti-HA IgD was expected to be an effective therapeutic treatment against influenza by IgD-stimulated basophils. We succeeded to induce all isotypes of the anti-HA antibodies in serum by hydrodynamic injection with each the plasmid vector coding antibody gene [21]. Moreover, we demonstrated that, in addition to anti-HA IgG, anti-HA IgA could also treat mice that were inoculated with the antibody gene 8 h after upper respiratory infection (Figure 5B) [21]. The viral titer was significantly decreased to almost undetectable levels by anti-HA IgA. Unexpectedly, the other isotypes failed to offer protection in our experiments [21].

## 3. Perspectives of Passive Immunization with Antibody Genes for Clinical Trials 

These pre-clinical studies have succeeded in the long-prophylaxis [6,11,20,46] and therapeutic treatment [21] of influenza virus infection with a single inoculation of the antibody gene. However, there are some issues that apply to antibody gene transfer for clinical trials. 

The first challenge is inducing potent expression of neutralizing mAbs in human subjects by antibody gene transfer at the same levels as seen in the pre-clinical studies. It is unknown whether the expression levels would be enough to offer protection from influenza virus infection [17]. From the study of Fabre et al., it can be speculated that the induction level of the transgene depends on animal species [70]. The expression levels in pig (18–20 kg weight) by hydrodynamic injection were ~200-fold lower than that in rats [70]. In a Phase I trial in 2000, Alvarez et al. were successful in inducing the expression of anti-HER2 by a single administration of an adenovirus vector encoding the antibody gene [71]. However, the therapeutic effect was not indicated in their report [71]. Moreover, there has been no follow-up clinical study [17]. Khorsandi et al. conducted the hydrodynamic injection in cirrhotic patients with thrombocytopenia [72]. They injected a plasmid encoding human thrombopoietin in human patients [72]. No medical benefit in the patients was found in this study, although experiments using the same method showed some promise in pigs [72]. It is possible that the expression of thrombopoietin was not sufficient to increase platelet numbers in the patients. 

Secondly, there is no way to terminate the expression of the neutralizing mAbs by the induction. It is necessary to find a way to regulate the expression level of the neutralizing mAbs by antibody gene transfer in humans. If an unwanted response against the mAbs were to occur in the patient, there would be no way to “turn off” the expression of the antibody gene. To address this problem, Hollevoet et al. reported an approach using an ecdysteroid-inducible gene expression system [17]. Ecdysteroids, such as ecdysone, are hormones that have been identified in plants, insects, and other related invertebrates [73]. The modified transgene turns on in the presence of ecdysteroid and is not active in its absence [73,74,75]. For a clinical approach, Cai et al. evaluated the pharmacokinetics of the synthetic analog of ecdysone, veledimex, in healthy human subjects in 2017 [76]. However, it seems that no clinical study has reported on the gene-transfer system under administration with veledimex as of yet [76]. Considering these facts, it is important to continuously develop more innovative medical devices or gene-expression systems to apply the antibody gene transfer method to human subjects. 

Finally, the third problem is the increase of the immunogenicity against the vector or expressed mAbs [77,78,79]. Although the gene transfer method with AAV continues to be used in clinical trials [80,81,82], Colella et al. have reported the immunogenicity of AAV-mediated gene therapy [81]. Unwanted immune responses to the antibodies derived from the AAV vector are induced in non-human primates, which result in decreased levels of delivered antibody [78]. Fuchs et al. reported that CD4-positive T cells may cause unwanted responses against idiotypic variations in the mAbs [78]. The AAV vector encoding the simian or simianized antibody gene has also induced anti-antibody responses to the AAV-delivered mAb in rhesus macaques [83]. The anti-antibodies mainly respond to variable regions of the mAb derived from the AAV vector [83]. Hollevoet et al. described a risk for increased immunogenicity of the mAbs that may be caused by the differences between natural antibody-producing cells and transfected cells (e.g., muscle, liver) [17]. Shimizu et al. discovered a new resident endoplasmic reticulum (ER) protein which folds and assembles immunoglobulin in plasma cells [84]. It is possible that there are differences between the post-translational modifications of the original antibodies and those of foreign mAbs derived from the antibody gene. These differences may cause antigenicity. However, we believe that it is not necessary to consider such a possibility, because the expression of mAbs derived from cells transfected with the antibody gene was sustained for a long time, as described above. Moreover, Johnson et al. successfully induced 200–300 µg/mL neutralizing mAbs between 8 and 12 months after transfer with the scAAV vector into rhesus macaques in the absence of immune suppression agents, such as cyclosporine [85]. Harding et al. reported that some human antibodies (e.g., golimumab and adalimumab) have the potential to induce an anti-idiotype antibody response in human patients [86]. They also reported that the induction of anti-golimumab antibodies correlates with reduction of circulating antibody [86]. However, an anti-idiotype antibody response induced by antibody gene transfer would be negligible, because no obvious decline in neutralizing activity has been observed long-term [11,20,50,51,85,87]. 

## 4. Conclusions 

From these pre-clinical studies for influenza [6,11,20,21,46], antibody gene-based injection using a plasmid or AAV vector is a possible method to induce a potent level of neutralizing anti-HA antibodies and maintain stable expression in human subjects. HA has much diversity among influenza virus strains [28]. Antigenic drift and shift often allow the virus to escape host immunity. Therefore, the broadly neutralizing anti-HA antibody [14] can be effective against both epidemic and pandemic influenza, even with only a single inoculation with the antibody gene [11,46]. Seasonal influenza virus initially infects the epithelial cells in the upper respiratory tract. Therefore, it is important to induce the neutralizing anti-HA antibody by antibody gene transfer in the upper respiratory tract to block the viral infection [6,20,21,46]. Gene-based transfer methods that induce neutralizing anti-HA mAbs can provide the benefits of lower costs and labor. Several researches also succeeded to neutralize other pathogens (e.g., human immunodeficiency virus (HIV), dengue virus, and Ebola virus) [17]. Innovation of antibody gene transfer methods for clinical applications may provide novel passive immunotherapies to protect against many infectious diseases.
